# Management practice and discharge outcome of patients with psychiatric disorder admitted to psychiatry wards of selected specialized settings in Ethiopia

**DOI:** 10.1186/s12888-023-04860-3

**Published:** 2023-05-17

**Authors:** Mubarik Fetu Semman, Fitsum Gezahegn Dadi, Girma Mamo Ijigu, Biruk Tafese Moges, Behailu Terefe Tesfaye

**Affiliations:** 1 Department of Pharmacy, College of Medicine and Health Sciences, Wolkite University, Wolkite, Ethiopia; 2 Department of Clinical Pharmacy, School of Pharmacy, College of Medicine and Health Sciences, Hawassa University, Hawassa, Ethiopia; 3 Department of Clinical Pharmacy, School of Pharmacy, Faculty of Health Science, Institute of Health, Jimma University, Jimma, Ethiopia; 4 Department of Pharmacy, College of Health Sciences, Mettu University, Mettu, Ethiopia

**Keywords:** Mental disorders, Treatment practice, Outcome, Risk factors, Ethiopia

## Abstract

**Background:**

Evidence on treatment practice, discharge outcomes, and associated factors in patients with psychiatric disorders are rarely discussed in Ethiopia. Results from the available studies are also seldom consistent and miss important factors, including treatment-related variables. Therefore, this study intended to describe management practice and discharge outcome among adult psychiatric patients admitted to psychiatry wards of selected specialized settings in Ethiopia. By pointing out associated factors, this study will also provide insight on targets to improve discharge outcomes.

**Patients and methods:**

A cross-sectional study was conducted involving 278 adult psychiatry patients admitted to the psychiatry wards of Jimma Medical Center and St. Amanuel Mental Specialized Hospital in the study period from December 2021 to June 2022. The data was analyzed using STATA V.16. Descriptive statistics and logistic regression analysis were performed to present patient characteristics and identify factors associated with discharge outcome, respectively. In all the analysis, *p* value < 0.05 was used to declare statistical significance.

**Results:**

Schizophrenia (125, 44.96%) and bipolar disorders (98, 35.25%) were the top two psychiatric disorders diagnosed at admission. A greater share of patients with schizophrenia were treated with the combination of diazepam, haloperidol, and risperidone than with diazepam and risperidone, 14 (5.04%) each. Patients with bipolar disorder were being treated primarily with the combination of diazepam, risperidone, and sodium valproate, or risperidone and sodium valproate, 14 (5.04%) each. Overall, 232 (83.4%) patients were on psychiatric polypharmacy. In this study, 29 (10.43%) patients were discharged unimproved, and this risk was significantly higher in those patients with a khat chewing habit (AOR = 3.59, 95% CI = 1.21–10.65, *P* = 0.021) than non-chewers.

**Conclusion:**

Psychiatric polypharmacy was found to be a common treatment approach in patients with psychiatric disorders. In the study, a little more than one-tenth of patients with psychiatric disorders were discharged without improvement. Hence, interventions targeting risk factors, especially khat use, should be undertaken to improve discharge outcomes in this population.

**Supplementary Information:**

The online version contains supplementary material available at 10.1186/s12888-023-04860-3.

## Introduction

Mental disorders are syndromes characterized by clinically significant disturbances in an individual’s cognition, emotional regulation, or behavior. These disturbances are associated with dysfunctions in the psychological, biological, or developmental processes [1], leading to distress or impairment in important areas of functioning [2]. Mental disorders include anxiety, depression, bipolar disorder, schizophrenia and other psychoses, attention deficit hyperactivity disorder (ADHD), obsessive–compulsive disorder, dementia, developmental disorders including autism, and alcohol and drug dependency [3].

Globally, in 2016, more than one billion people were affected by mental or addictive disorders, accounting for approximately 16% of the global population. While the global data in 2017 shows an estimated 264 million, 45 million, and 20 million people were living with depression, bipolar disorder, and schizophrenia, respectively [4, 5]. In Africa, including Ethiopia, nearly similar proportions have been reported from different parts of the continent [6–8].

Depending on their presentations, patients with psychiatric disorders can be treated either in an outpatient or inpatient setting. Inpatient treatment is chosen particularly when the patient behaves disorganized, exhibits psychotic features, or cannot be safely managed as an outpatient [9]. The treatment depends on the type of mental illness, its severity, and what works best for the patient and may include medication such as antipsychotics, antidepressants, mood stabilizers, psychotherapy, alternative therapies, or brain stimulation therapy. In many cases, patients respond better to a combination of treatments [10, 11].

The quality of mental health services varies across the globe. For instance, the existing mental health services in low-income countries have been characterized as inadequate, inequitable, and inefficient. In developing nations, particularly in Sub-Saharan Africa, the lack of infrastructure and the limited number of psychiatric beds per population remain a significant barrier to improving mental health services in the region [12–14]. This may negatively impact patient outcomes [13].

In general, there are only limited studies on the discharge outcomes of patients with psychiatric disorders. According to the available research, a high proportion of discharges results in a better outcome [15, 16]. For instance, in one retrospective cross-sectional study from Nepal involving 3687 patients with psychiatric disorders, improved discharge outcomes were recorded in 92% of the patients, and three patients died in the ward [15]. In another retrospective study carried out in Malawi, including 417 hospitalized patients with psychiatric illness, it was reported that 283 (68.03%) of patients were stabilized and discharged home, 81 (19.47%) patients were transferred to another hospital, 34 (8.17%) patients were discharged against medical advice, and 14 (3.37%) of the patients absconded. Four (0.96%) of the patients died in the hospital [16].

In Ethiopia, evidence on treatment practices, outcomes, and associated factors in patients with psychiatric disorders is rarely discussed in the literature. Results from the available studies are also seldom consistent. The existing studies reported discharge with improvement of 90.3% [12] and 74.9% [17], respectively. Only one of the studies addressed associated factors, and being married, better educated, and having a longer hospital stay predicted a better outcome [17]. However, this study missed important factors, including treatment-related variables. The available studies are also retrospective, having been conducted a half decade ago, and they are single-centered. Therefore, the present study has addressed the treatment practice, discharge outcomes, and associated factors in patients with psychiatric disorders admitted to two specialized psychiatry settings, considering additional independent factors.

## Patients and methods

### Study setting and periods

The study was conducted from December 1, 2021, to June 30, 2022, at the Psychiatry Ward of Jimma Medical Center (PWJMC) and St. Amanuel Mental Specialized Hospital (SAMSH). SAMSH is the largest mental specialized hospital in Ethiopia, providing mental health services to clients from all over the country. It is located in Addis Ababa, the capital of Ethiopia. At the outpatient level, it serves more than 800 patients daily. It has a capacity of 270 beds for inpatient care. The number of emergency visits per month is close to 2000 [18, 19]. On the other hand, JMC is located in Jimma Town, 352 km southwest of Addis Ababa, Ethiopia. It is the only teaching, referral, and medical center in the south-western part of the country, with a bed capacity of 800. According to JMC statistics, the center currently serves approximately 15,000 inpatients and 160,000 outpatients each year, with a catchment population of approximately 15 million people. One of the units is the psychiatry department, which was established in 1988 and is next to St. Amanuel mental specialized hospital. Currently, there are approximately 5405 patients on follow-up, and the clinic officially has 53 inpatient beds [20].

### Study design

A cross-sectional study was conducted involving adult psychiatry patients admitted to PWJMC and SAMSH.

### Population

#### Source population

All adult psychiatry patients admitted to PWJMC and SAMSH.

#### Study population

All adult psychiatry patients admitted to PWJMC and SAMSH who fulfilled the inclusion criteria during the study period.

### Inclusion and exclusion criteria

#### Inclusion criteria

All psychiatric patients age ≥ 18 years who were admitted to PWJMC and SAMSH during the study period.

#### Exclusion criteria

Those who were refused to participate.

Patients who stayed in hospital beyond the study period.

Patients who were unable to communicate.

### Sample size and sampling technique

The sample size for patients’ discharge outcome was calculated using a single population proportion formula. A 50% proportion (P) was considered for patients’ unimproved discharge outcome. Considering a 0.05 margin of error (d) and 95% confidence interval, n = the required sample size.

$$\mathrm n=\frac{\left(\mathrm{Za}/2\right)^2\mathrm p\left(1-\mathrm p\right)}{\mathrm d^2}$$

p = Assumed proportion of patients’ unimproved discharge outcome = 0.5

1-p = q = 0.5

d = Expected margin of error = 0.05

Z α/2 = 95%confidence interval (C.I) = 1.96

Thus, n = ((1.96)2 × 0.5x 0.5)/ (0.05)^2^ = 384

Since the target population was less than 10,000, the sample size should be corrected using the following correction formula.

$$\mathbf{nf}\boldsymbol=\mathbf{No}\boldsymbol/\mathbf1\boldsymbol+\mathbf{No}\boldsymbol/\mathbf N$$

Where nf is the corrected sample size and N is the number of patients admitted in the two hospitals in the last years’ seven month period of similar season, which was 833.

Thus, nf = 384/1 + 384/833 = 264.

After accounting for a 5% non-response (14 patients), the final sample size was 278 patients

Based on previous admission data, this number was proportionally divided in the ratio of 1:5 for both hospitals. Accordingly, 47 and 231 patients were allocated for PWJMC and SAMSH, respectively.

A consecutive sampling technique was used to recruit the study participants.

### Data collection instrument and processing

Data was collected through patient interviews and a review of medical records using a questionnaire developed after reviewing relevant literature [12, 15–17]. For patient interviews, the questionnaire was translated into the most common local languages (Afan Oromo and Amharic). Socio-demographic and behavioral, clinical, drug, substance abuse, and treatment-related information were collected. The diagnosis of psychiatric illnesses and the assessment of outcome at discharge were made using the Diagnostic and Statistical Manual of Mental Disorders, Fifth Edition (DSM-5), and the Clinical Global Impression (CGI) scale, respectively, by a psychiatrist or a senior psychiatric nurse who are not the authors of this article.

The Life Events Questionnaire (LEQ) was used to assess recent life events. It is a 12-item self-rating instrument that assesses common life events that are potentially threatening. Respondents were asked to select life events that had occurred within the past 12 months prior to the onset of their psychiatric symptoms. Each life event was answered dichotomously (yes or no) and was scored 1 if it was ‘yes’ and 0 if it was "no." A total score was the sum of all items [21].

The Oslo Social Support Scale (OSSS-3) was used to assess perceived psychosocial support. The OSSS-3 consists of three items that assess the level of social support. The sum score ranges from 3 to 14, with high values representing strong levels of social support and low values representing poor levels of social support, which was interpreted as [3-8] is poor social support, [9-11] is moderate social support, and [12-14] is strong social support [22].

The adherence assessment tool, the Medication Adherence Rating Scale (MARS-5), was used to assess adherence. MARS-5 is a 5-item self-report scale that is used to detect non-adherent behavior by self-report. It is a measure of non-adherence in general, not for mental disorders in particular. The questions are formulated in a non-threatening and non-judgmental way to minimize social desirability bias. The item responses are scored on a 5-point Likert scale, where 1 = always, 2 = often, 3 = sometimes, 4 = rarely, and 5 = never. Scores range from 5 to 25, with higher scores indicating higher adherence. Psychiatric patients who scored 23 or above were classified as adherent to their psychotropic medication, while those who scored less than 23 were classified as non-adherent [23, 24].

The Mental Health Trigger Tool (MHTT) was used for efficient chart review and identification of adverse drug reactions (ADR) in addition to self-reported ADR by patients themselves. The MHTT was developed with the aim of detecting and measuring both traditionally defined ADRs and other patient safety incidents relevant to mental health settings. It is an easy-to-use tool for understanding and measuring a variety of patient safety incidents in mental health settings and it is designed for use in inpatient mental health settings. The tool contains a list of 25-item triggers related to general care, laboratory, medication-related, and behavior-related items [25].

The clinical global impression (CGI) scale was used to assess the outcome at discharge. The CGI is an overall clinician-determined summary measure that considers all available information, including knowledge of the patient’s history, psychosocial circumstances, symptoms, behavior, and the impact of the symptoms on the patient’s ability to function. It has two components: the CGI-Severity (CGI-S), which rates illness severity, and the CGI-Improvement (CGI-I), which rates change from the initiation (baseline) of treatment. The CGI-S asks the clinician one question: "Considering your total clinical experience with this particular population, how mentally ill is the patient at this time?" which is rated on the following seven-point scale: 1 = normal, not at all ill; 2 = borderline mentally ill; 3 = mildly ill; 4 = moderately ill; 5 = markedly ill; 6 = severely ill; 7 = among the most extremely ill patients. The CGI-I is similarly simple in its format. Each time the patient is seen after treatment has been initiated, the clinician compares the patient’s overall clinical condition to the one-week period just prior to the initiation of treatment (during admission). The CGI-S score obtained at the baseline visit serves as the basis for this assessment. Again, only the following query is rated on a seven-point scale: "Compared to the patient’s condition at admission, this patient’s condition is: 1 = very much improved since the initiation of treatment; 2 = much improved; 3 = minimally improved; 4 = no change from baseline; 5 = minimally worse; 6 = much worse; 7 = very much worse since the initiation of treatment." There are no universally accepted scoring guidelines for the seven anchor points; rather, they were designed to be based solely on clinical judgment. The CGI is applicable across all CNS studies, including depression, schizophrenia, bipolar disorder, and anxiety, no matter the population, drug, or other main study measures. It provides a readily recognizable and universally known efficacy measure that distinguishes it from the more complex, lengthier, and sometimes difficult to administer efficacy scales [26].

Three data collectors were trained on the research tool and data collection procedure. All patients included in the study were followed from the first day in the psychiatry ward until the date of discharge using a follow-up chart included in the questionnaire.

### Data quality assurance

The questionnaire was carefully tailored to collect all of the necessary information, and it was translated from English to Afan Oromo and Amharic and then back translated into English. A pretest was conducted on 5% of the study participants from JMC. Three trained psychiatry nurses and clinical pharmacists collected the data. After data was collected, before being exported to STATA V.16 for analysis, the data was cleared, categorized, compiled, coded, and also checked for completeness and accuracy.

### Data processing and statistical analysis

Epidata V. 4.2.0 was utilized for data entry, and the data was exported to STATA V.16 for analysis. Continuous variables were summarized using the mean ± standard deviation (SD), while categorical data were reported as frequencies and percentages. A chi-square test was performed to check the adequacy of cells before conducting regression. To examine multicollinearity, the variance inflation factor (VIF) was assessed, and independent variables with a VIF < 6 were included in the model. For the discharge outcome, bivariate logistic regression was used to identify candidate variables for multivariate logistic regression. Variables with a *p*-value < 0.25 in bivariate regression were considered suitable for multivariate logistic regression. Then, multivariate logistic regression was employed to identify independent predictors of an unimproved discharge outcome. The odds ratio was used as a measure of the strength of association and *p*-value < 0.05 was considered to declare statistical significance. The Hosmer and Lemeshow test (*p* > 0.05) was performed, indicating good fit.

### Operational definition and definition of terms

#### Patients discharge outcome

Condition of the patient at discharge compared to the patient’s condition at admission. Based on the CGI score and for the convenience of this study the patient’s discharge outcome was classified as ‘currently improved’ if the patient’s condition at discharge was very much improved; much improved or minimally improved since admission. Similarly, the patient’s discharge outcome was classified as ‘currently not improved’ if the patient’s condition at discharge was no change from baseline; minimally worse; much worse; very much worse since the admission, left against medical advice, died, referred or absconded [26].

#### Medical comorbidity

A medical condition in a patient that causes, is caused by, or is otherwise related to another condition in the same patient [27].

#### Psychiatric polypharmacy

The prescription of two or more psychotropic medications concurrently to a patient [28].

#### Substance use

Use of substance(s) on a consistent and habitual basis for a period of more than one month [29]. Substances include psychoactive substances such as alcohol, khat (Catha edulis), cigarette, and cannabis.

#### Substance abuse

A maladaptive pattern of substance use leading to clinically significant impairment or distress as manifested by one (or more) of the following occurring within a 12-month period: recurrent substance use resulting in a failure to fulfill major role obligations at work, school, or home, recurrent substance use in situations in which it is physically hazardous, recurrent substance-related legal problems, and continued substance use despite having persistent or recurrent social or interpersonal problems caused or exacerbated by the effects of the substance [30].

## Results

### Participant enrolment

**Fig. 1:**
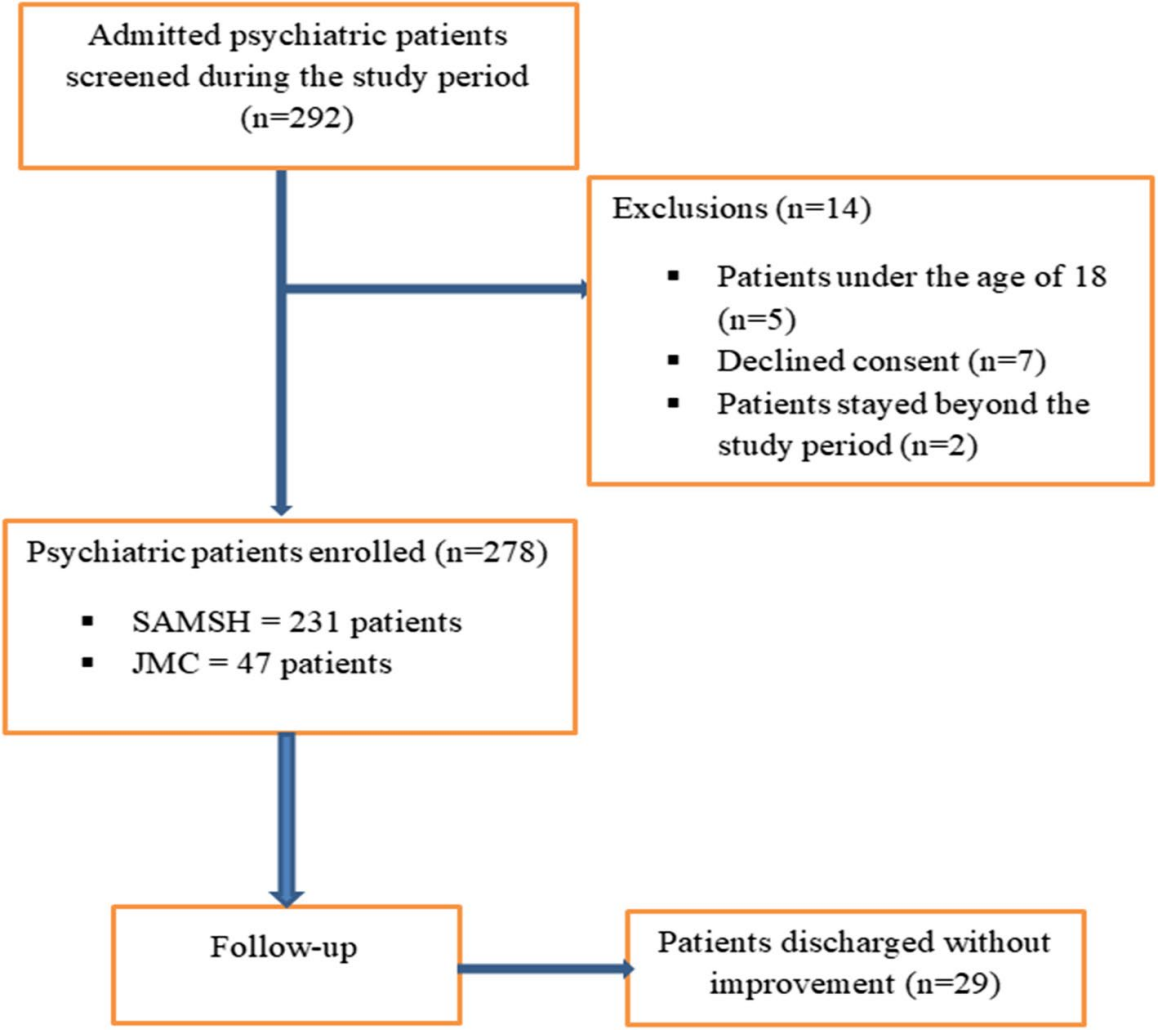
Study participant’s enrollment at PWJMC and SAMSH from December 2021 to June 2022. SAMSH = SAMSH = St. Amanuel mental specialized hospital, JMC = Jimma medical center

Among the 292 admitted psychiatric patients screened during the study period, 278 patients fulfilled the inclusion criteria. Fourteen patients were excluded from enrolment for not meeting inclusion criteria, with a response rate of 95.2% **(**Fig. 1**)**.

### Socio-demographic and behavioral characteristics

Among the 278 patients enrolled, 165 (59.35%) were male, and the mean ± standard deviation of the age of the patients was 32.3 ± 9.7 years. Self-reported substance use was noted in 113 (40.65%) of the study participants. More than one third, 106 (38.13%) of the patients, had family history of psychiatric  illnesses. With regard to social support, 172 (61.87%) and 47 (16.91%) of the patients reported moderate and poor social support, respectively **(**Table 1**)**.

**Table 1:** Socio-demographic characteristics of study participants at PWJMC and SAMSH from December 2021 to June 2022

Variables, N (278)	Frequency	Percent
Age of participants (years), μ and SD	32.3 ± 9.7	
Age group		
18–30	136	48.92
31–45	117	42.09
46–65	25	8.99
Sex		
Male	165	59.35
Female	113	40.65
Marital status		
Single	166	59.71
Married	83	29.86
Divorced	27	9.71
Widowed	2	0.72
Living situation		
Alone	23	8.27
With partner or family	255	91.73
Residence		
Urban	165	59.35
Rural	113	40.65
Educational status		
Not able to read and write	38	13.67
Informal education	3	1.08
Primary school	76	27.34
High school	95	34.17
University or college	66	23.74
Occupational status		
Farmer	49	17.63
Merchant	13	4.68
Civil servant	45	16.19
Self employed	40	14.39
Daily labor	10	3.6
Has no job	121	43.53
Average monthly income (Birr), μ and SD	3830.6 ± 5746.5	
Family history of psychiatric illnesses		
Yes	106	38.13
No	172	61.87
Social support status		
Poor social support	47	16.91
Moderate social support	172	61.87
Strong social support	59	21.22

*PWJMC* psychiatry ward of Jimma medical center, *SAMSH* St. Amanuel mental specialized hospital,* μ ± SD* mean and standard deviation

### Clinical characteristics

Clinically, the majority of the study participants were known psychiatric patients: 204 (73.38%). More than half of the enrolled participants had previous history of hospitalization, 151 (54.32%). More than a quarter, 74 (26.62%) of patients, had a suicide risk at admission. The mean ± standard deviation of the hospital length of stay of the patients was 40.9 ± 20.7 days **(**Table 2**)**.

**Table 2:** Clinical characteristics of study participants at PWJMC and SAMSH from December 2021 to June 2022

Variables, N (278)	Frequency	Percent
Medical comorbidities		
Present	37	13.31
Absent	241	86.69
Specific medical comorbidities		
Human immunodeficiency virus disease	11	3.96
Hypertension	11	3.96
Epilepsy	8	2.88
Peptic ulcer disease	7	2.52
Asthma	2	0.72
Blindness	1	0.36
Types of psychiatric diagnosis		
Newly diagnosed psychiatric patient	74	26.62
Known psychiatric patient	204	73.38
Duration since diagnosis (years), μ and SD	5.7 ± 7.1	
Previous hospitalization history		
Yes	151	54.32
No	127	45.68
Number of previous admission		
No previous admission	127	45.68
1 time	61	21.94
2 time	40	14.39
≥ 3 times	50	17.99
Duration since last admission (months)		
No previous admission	127	45.68
≤ 12	47	16.91
13–24	42	15.11
> 24	62	22.3
History of life threatening events		
No life event	106	38.13
At least one life event	172	61.87
Involuntary admission		
Yes	214	76.98
No	64	23.02
History of violence at admission		
Yes	199	71.58
No	79	28.42
Specific type of violence		
No violence	79	28.42
Deliberate self-harm	22	7.91
Harm to others	55	19.78
Harm to property	17	6.12
More than one type	105	37.77
Suicide risk at admission		
Yes	74	26.62
No	204	73.38
Types of discharge from current admission		
Based on psychiatrist recommendation	260	93.53
Premature discharge	18	6.47
Hospital length of stay (days), μ and SD	40.9 ± 20.7	
Place of follow up after discharge		
Hospital	275	98.92
Health center/ clinic	2	0.72
No follow up	1	0.36
Outpatient contact with psychiatrist		
Yes	263	94.6
No	15	5.4
Pattern of follow up		
Regular	222	79.86
Irregular	56	20.14

### Psychiatric disorders diagnosed at admission

**Fig. 2:**
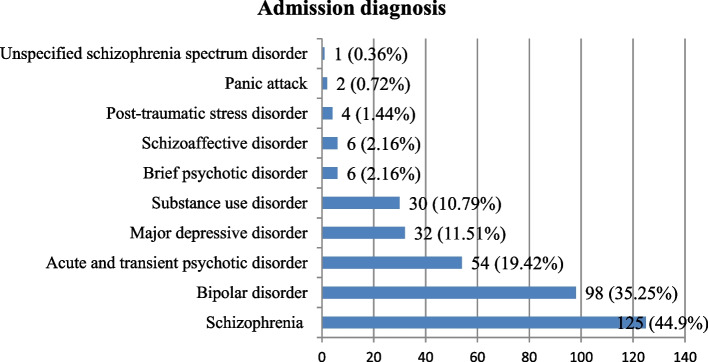
Admission diagnosis of study participants at PWJMC and SAMSH from December 2021 to June 2022

Schizophrenia, 125 (44.96%), and bipolar disorders, 98 (35.25%), were the top two admission diagnoses followed by acute and transient psychotic disorder, 54 (19.42%), major depressive disorder, 32 (11.51%) and Substance use disorders, 30 (10.79) respectively (Fig. 2).

### Treatment related characteristics

In terms of pharmacotherapy, 274 (98.56%) of the patients received at least one psychotropic medication during their hospital stay and at discharge. Among the conventional antipsychotics, haloperidol was the most widely used drug, 65 (23.38%). More than two thirds, 192 (69.06%) of the study participants were prescribed risperidone. Sodium valproate was the most commonly used, 79 (28.42%), drug among the mood stabilizers. Fluoxetine took a greater share of prescriptions, 21 (7.55%), among anti-depressants. Nearly half of the patients enrolled, 123 (44.24%), received diazepam. Trihexyphenidyl, 34 (12.23%), was the most commonly used non-psychotropic medication.

Psychiatric polypharmacy was observed in a greater proportion, 232 (83.45%), of study participants. Nearly three-quarters of the patients, 205 (73.74%), had psychotropic drug use history. More than half of the study participants, 144 (51.8%) were non-adherent towards their medications. Electroconvulsive therapy was instituted in only 20 (7.19%) patients (Table 3).

**Table 3:** Treatment related characteristics of study participants at PWJMC and SAMSH from December 2021 to June 2022

Variables, N (278)	Frequency	Percent
Category of psychotropic medications used		
Conventional antipsychotics		
Haloperidol	65	23.38
Flufenazine decanoate depot	21	7.55
Chlorpromazine	14	5.04
Atypical antipsychotics		
Risperidone	192	69.06
Olanzapine	35	12.59
Mood stabilizer		
Sodium valproate	79	28.42
Carbamazepine	22	7.91
Anti-depressant		
Fluoxetine	21	7.55
Amitriptyline	16	5.76
Imipramine	15	5.4
Sertraline	2	0.72
Benzodiazepines		
Diazepam	123	44.24
Lorazepam	22	7.91
Bromazepam	15	5.4
Non-psychotropic co-medications used		
Trihexyphenidyl	34	12.23
Thiamine	14	5.04
Propranolol	7	2.52
Phenobarbital	5	1.8
Phenytoin	2	0.72
HAART	5	1.8
Amlodipine	4	1.44
Salbutamol puff	2	0.72
Multivitamins	2	0.72
Number of psychotropic medications used per patient, μ and SD	2.31 ± 0.91	
Psychiatric polypharmacy		
Yes	232	83.45
No	46	16.55
Number of non-psychotropic medications used per patient, μ and SD	0.27 ± 0.50	
Total number of medications used per patient, μ and SD	2.57 ± 0.98	
Past psychotropic medication history		
Yes	205	73.74
No	73	26.26
Length of psychotropic treatment before admission (years), μ and SD	4.31 ± 5.78	
Electroconvulsive therapy use		
Yes	20	7.19
No	258	92.81
Self-reported substance use		
Yes	113	40.65
No	165	59.35
Specific substance use		
Khat	104	37.41
Cigarette	78	28.06
Alcohol	58	20.86
Cannabis	2	0.72
Adherence to medications		
Non-adherent	144	51.8
Adherent	134	48.2
Is there ADR?		
Yes	55	19.78
No	223	80.22
Specific ADR encountered		
Pseudo parkinsonism	35	12.59
Dystonia	14	5.04
Akathisia	6	2.16
Orthostatic hypotension	5	1.8
Weight gain	16	5.76

### Pharmacotherapy received per psychiatric diagnosis

The large majority of the study participants were being treated with combination pharmacotherapy. A greater share of the schizophrenic patients were treated with the combination of diazepam, haloperidol, and risperidone or diazepam and risperidone, 14 (5.04%) each. Bipolar patients were being treated mainly with the combination of either diazepam, risperidone, and sodium valproate or risperidone and sodium valproate, 14 (5.04%) each. The combination of fluoxetine and risperidone, 8 (2.88%) were the most commonly used drugs among major depressive disorder patients. Among patients with substance use disorder diazepam and risperidone, 6 (2.16%) were the most widely used drug combinations (Table 4).

**Table 4:** Treatment received per psychiatric diagnosis by study participants at PWJMC and SAMSH from December 2021 to June 2022

Treatment received per psychiatric diagnosis, N (%)						
Psychotropic drugs received by the patient	Schizophrenia	Bipolar disorders	MDD	Substance Use disorders	ATPD	Others
Amitriptyline	-	-	1 (0.36)	2 (0.72)	-	-
Amitriptyline, Chlorpromazine and Diazepam	-	-	2 (0.72)	-	2 (0.72)	-
Amitriptyline, Diazepam and Haloperidol	1 (0.36)	-	-	-	-	-
Amitriptyline, Diazepam, Haloperidol and Risperidone	1 (0.36)	-	-	-	-	-
Amitriptyline, Diazepam and Risperidone	-	-	2 (0.72)	-	2 (0.72)	-
Amitriptyline, Flufenazine decanoate and Olanzapine	2 (0.72)	-	-	-	-	-
Amitriptyline and Risperidone	4 (1.44)	-	4 (1.44)	-	2 (0.72)	-
Bromazepam, Diazepam, Haloperidol, Risperidone and Na + valproate	-	2 (0.72)	-	-	2 (0.72)	-
Bromazepam, Flufenazine decanoate and Olanzapine	2 (0.72)	-	-	-	-	-
Bromazepam and Haloperidol	2 (0.72)	-	-	-	-	-
Bromazepam, Imipramine and Olanzapine	-	-	2 (0.72)	-	1 (0.36)	-
Bromazepam and Risperidone	3 (2 (0.72)	-	-	-	-	-
Bromazepam Risperidone and Na + valproate	-	4 (1.44)	-	-	4 (1.44)	-
Carbamazepine and Chlorpromazine	-	2 (0.72)	-	-	1 (0.36)	-
Carbamazepine, Diazepam, Haloperidol and Risperidone	-	7 (2.52)	-	-	2 (0.72)	-
Carbamazepine, Diazepam and Risperidone	-	9 (3.24)	-	-	-	-
Carbamazepine and Haloperidol	2 (0.72)	-	-	-	-	-
Carbamazepine and Risperidone	2 (0.72)	-	-	-	-	-
Chlorpromazine	2 (0.72)	-	-	2 (0.72)	2 (0.72)	-
Chlorpromazine and Flufenazine decanoate	2 (0.72)	-	-	-	-	-
Chlorpromazine, Haloperidol and Na + valproate	-	2 (0.72)	-	-	-	-
Chlorpromazine and Risperidone	2 (0.72)	-	-	-	-	-
Diazepam	-	-	-	3 (1.08)	-	-
Diazepam, Flufenazine decanoate and Haloperidol	1 (0.36)	-	-	-	-	-
Diazepam, Flufenazine decanoate, Risperidone and Na + valproate	-	1 (0.36)	-	-	-	-
Diazepam, Fluoxetine, Lorazepam and Olanzapine	-	-	2 (0.72)	-	2 (0.72)	-
Diazepam, Fluoxetine and Risperidone	2 (0.72)	-	2 (0.72)	-	-	-
Diazepam, Fluoxetine, Risperidone and Na + valproate	-	-	-	-	-	2 (0.72)
Diazepam and Haloperidol	-	-	-	2 (0.72)	-	1 (0.36)
Diazepam, Haloperidol and Olanzapine	2 (0.72)	-	-	-	-	-
Diazepam, Haloperidol and Risperidone	14 (5.04)	2 (0.72)	-	-	-	2 (0.72)
Diazepam, Haloperidol, Risperidone and Na + valproate	1 (0.36)	6 (2.16)	-	-	1 (0.36)	-
Diazepam, Haloperidol and Na + valproate	2 (0.72)	7 (2.52)	-	-	-	-
Diazepam, Imipramine and Olanzapine	1 (0.36)	-	-	-	-	1 (0.36)
Diazepam, Imipramine Risperidone and Na + valproate	-	2 (0.72)	-	-	-	-
Diazepam, Lorazepam and Risperidone	-	-	-	-	-	2 (0.72)
Diazepam and Olanzapine	5 (1.80)	1 (0.36)	-	2 (0.72)	2 (0.72)	-
Diazepam, Olanzapine and Na + valproate	-	-	-	-	-	2 (0.72)
Diazepam and Risperidone	14 (5.04)	2 (0.72)	2 (0.72)	6 (2.16)	4 (1.44)	-
Diazepam, Risperidone and Na + valproate	1 (0.36)	14 (5.04)	-	-	6 (2.16)	2 (0.72)
Flufenazine decanoate	-	-	-	1 (0.36)	1 (0.36)	-
Flufenazine decanoate and Haloperidol	2 (0.72)	-	-	-	-	-
Flufenazine decanoate and Olanzapine	-	2 (0.72)	-	-	2 (0.72)	-
Flufenazine decanoate and Risperidone	5 (1.80)	-	-	2 (0.72)	1 (0.36)	-
Flufenazine decanoate, Risperidone and Na + valproate	-	3 (1.08)	1 (0.36)	-	1 (0.36)	-
Fluoxetine, Olanzapine and Na + valproate	-	2 (0.72)	-	-	-	-
Fluoxetine and Risperidone	2 (0.72)	2 (0.72)	8 (2.88)	2 (0.72)	5 (1.80)	2 (0.72)
Haloperidol	2 (0.72)	-	-	-	-	-
Haloperidol and Imipramine	2 (0.72)	-	2 (0.72)	-	-	-
Haloperidol and Risperidone	1 (0.36)	-	-	1 (0.36)	-	-
Haloperidol and Na + valproate	-	2 (0.72)	-	-	-	-
Imipramine and Risperidone	5 (1.80)	-	2 (0.72)	-	2 (0.72)	-
Lorazepam	-	2 (0.72)	-	1 (0.36)	-	-
Lorazepam and Risperidone	13 (4.68)	-	-	-	-	2 (0.72)
Olanzapine	4 (1.44)	2 (0.72)	2 (0.72)	-	2 (0.72)	-
Olanzapine and Na + valproate	-	4 (1.44)	-	-	3 (1.08)	-
Risperidone	13 (4.68)	-	-	2 (0.72)	-	-
Risperidone and Sertraline	2 (0.72)	-	-	-	-	2 (0.72)
Risperidone and Na + valproate	4 (1.44)	14 (5.04)	-	-	4 (1.44)	1 (0.36)
Na + valproate	-	4 (1.44)	-	-	-	-

### Discharge outcomes

Among the 278 patients followed, 29 patients (10.43%) were discharged without improvement (Table 5).

**Table 5:** Discharge outcomes of study participants at PWJMC and SAMSH from December 2021 to June 2022

Discharge outcomes N (278)	Frequency	Percent
Condition of patients at discharge		
Very much improved	4	1.44
Much improved	161	57.91
Minimally improved	84	30.22
Same (no change)	11	3.96
Minimally worse	3	1.08
Much worse	1	0.36
Left against medical advice	9	3.24
Absconded	5	1.80
Condition of patients at discharge summary		
Currently improved	249	89.57
Currently not improved	29	10.43

### Strength of association between covariates and discharge outcomes among study participants

In a binary logistic regression, from the socio-demographic characteristics, discharge outcome was significantly associated with age (*p* = 0.033) and rural residence (*P* = 0.041) **(**Table 6). Whereas, no clinical characteristics variable was significantly associated with an unimproved discharge outcome (Table 7).

**Table 6:** Crudes and adjusted odds ratio (OR) of socio-demographic characteristics and discharge outcome among study participants at PWJMC and SAMSH from December 2021 to June 2022

Variable, N (278)	Discharge outcomes		COR (95%CI)	*P*-Value	AOR (95%CI)	*P*-Value
	Improved n (%)	Not improved, n (%)				
Age of participants, μ and SD	32.3 ± 9.7		1.04 (1.00–1.08)	**0.033**	1.01(0.96–1.07)	0.579
Sex						
Male	146 (52.52)	19 (6.83)	1			
Female	103 (37.05)	10 (3.60)	0.74 (0.33–1.67)	0.476	-	
Living situation						
Alone	20 (7.19)	3 (1.08)	1.32 (0.37- 4.74)	0.67	-	
With partner or family	229 (82.37)	26 (9.35)	1			
Residence						
Urban	153 (55.04)	12 (4.32)	1		1	
Rural	96 (34.53)	17 (6.12)	2.26 (1.03–4.93)	**0.041**	2.15 (0.81–5.71)	0.126
Employment						
Employed	135 (48.56)	20 (7.19)	1		1	
Not employed	114 (41.01)	9 (3.24)	0.53 (0.23–1.22)	0.135	0.37 (0.14–1.03)	0.056
Average monthly income (Birr), μ and SD	3830.6 ± 5746.5		0.99 (0.99- 1.00)	0.297	-	
Family history of psychiatric illnesses						
Yes	95 (34.17)	11 (3.96)	0.99 (0.45–2.18)	0.981	-	
No	154 (55.40)	18 (6.47)	1

**Table 7:** Crudes and adjusted odds ratio (OR) of clinical characteristics and discharge outcome among study participants at PWJMC and SAMSH from December 2021 to June 2022

Variable, N (278)	Discharge outcomes		COR (95%CI)	*P*-Value	AOR (95%CI)	*P*-Value
	Improved, n (%)	Not improved, n (%)				
Schizophrenia						
Yes	109 (39.21)	16 (5.76)	1.58 (0.73- 3.42)	0.246	0.90 (0.34–2.36)	0.833
No	140 (50.36)	12 (4.68)	1		1	
Bipolar disorder						
Yes	88 (31.65)	10 (3.60)	0.96 (0.42–2.16)	0.927	-	
No	161 (57.91)	19 (6.83)	1			
Major depressive disorder						
Yes	31 (11.15)	1 (0.36)	0.25 (0.032—1.91)	0.182	0.38 (0.33–4.48)	0.445
No	218 (78.42)	28 (10.07)	1		1	
Substance use disorder						
Yes	29 (10.43)	1 (0.36)	0.27 (0.035–2.07)	0.208	0.23 (0.02–2.33)	0.214
No	220 (79.14)	28 (10.07)	1		1	
Acute and transient psychotic disorder						
Yes	50 (17.99)	4 (1.44)	0.64 (0.21—1.91)	0.421	-	
No	199 (71.58)	25 (8.99)	1			
Medical comorbidities						
Present	34 (12.23)	3 (1.08)	0.73 (0.21- 2.54)	0.621	-	
Absent	215 (77.34)	26 (9.35)	1			
Types of psychiatric diagnosis						
Newly diagnosed	70 (25.18)	4 (1.44)	1		1	
Known	179 (64.39)	25 (8.99)	2.44 (0.82–7.27)	0.108	4.18 (0.42–41.96)	0.224
Duration since diagnosis (years), μ and SD	5.7 ± 7.06		1.05 (0.99—1.09)	0.067	0.97 (0.83–1.14)	0.737
Previous hospitalization history						
Yes	133 (47.84)	18 (6.47)	1.43 (0.65—3.15)	0.378	-	
No	116 (41.73)	11 (3.96)	1			
History of life threatening events						
No life event	92 (33.09)	14 (5.04)	1		1	
At least one life event	157(56.47)	15 (5.40)	0.62 (0.29–1.36)	0.238	0.41 (0.16–1.05)	0.064
Involuntary admission						
Yes	190 (68.35)	24 (8.63)	1.49 (0.54- 4.08)	0.437	-	
No	59 (21.22)	5 (1.80)	1			
History of violence at admission						
Yes	175 (62.95)	24 (8.63)	2.03 (0.75–5.52)	0.166	0.94(0.29–3.08)	0.917
No	74 (26.62)	5 (1.80)	1		1	
Suicide risk at admission						
Yes	62 (22.30)	12 (4.32)	2.13 (0.96–4.70)	0.062	2.51 (0.94–6.68)	0.065
No	187 (67.27)	17 (6.12)	1		1	
Hospital length of stay (days), μ and SD	40.9 ± 20.69		0.99 (0.97- 1.01)	0.579	-	

From the treatment related characteristics, discharge outcome was significantly associated with being on haloperidol (*p* = 0.019) and the length of psychotropic treatment before admission (*p* = 0.025). However, after adjusting for variables with a *p*-value < 0.25, multivariate logistic regression analysis identified being a khat user as the only independent predictor of an unimproved discharge outcome. Accordingly, khat users were about 3.6 times more likely to be discharged without improvement than patients who were non khat users (AOR: 3.59, 95% CI: 1.21–10.65, *P* = 0.021) (Table 8).

**Table 8:** Crudes and adjusted odds ratio (OR) of treatment related characteristics and discharge outcome among study participants at PWJMC and SAMSH from December 2021 to June 2022

Variables, N (278)	Discharge outcomes		COR(95%CI)	*P*-Value	AOR(95%CI)	*P*-Value
	Improved, n (%)	Not improved, n (%)				
Haloperidol						
Yes	53 (19.06)	12 (4.32)	2.61 (1.17–5.80)	**0.019**	1.89 (0.70–5.06)	0.207
No	196 (70.50)	17 (6.12)	1		1	
Flufenazine decanoate depot						
Yes	19 (6.83)	2 (0.72)	0.90 (0.20–4.06)	0.887	-	
No	230 (82.73)	27 (9.71)	1			
Risperidone						
Yes	170 (61.15)	22 (7.91)	1.46 (0.60- 3.56)	0.405	-	
No	79 (28.42)	7 (2.52)	1			
Olanzapine						
Yes	33 (11.87)	2 (0.72)	0.48 (0.11—2.13)	0.338	-	
No	216 (77.70)	27 (9.71)	1			
Sodium valproate						
Yes	71 (25.54)	8 (2.88)	0.96 (0.40—2.26)	0.917	**-**	
No	178 (64.03)	21 (7.55)	1			
Carbamazepine						
Yes	21 (7.55)	1 (0.36)	0.39 (0.05- 2.99)	0.364	-	
No	228 (82.01)	28 (10.07)	1			
Fluoxetine						
Yes	18 (6.47)	3 (1.08)	1.48 (0.41- 5.37)	0.550	-	
No	231 (83.09)	26 (9.35)	1			
Diazepam						
Yes	109 (39.21)	14 (5.04)	1.20 (0.55–2.59)	0.645	-	
No	140 (50.36)	15 (5.40)	1			
Lorazepam						
Yes	19 (6.83)	3 (1.08)	1.40 (0.39–5.04)	0.610	-	
No	230 (82.73)	26 (9.35)	1			
Past psychotropic medication history						
Yes	181 (65.11)	24 (8.63)	1.80 (0.66- 4.92)	0.249	0.15 (0.01–1.46)	0.102
No	68 (24.46)	5 (1.80)	1		1	
Length of psychotropic treatment (years), (μ and SD	4.31 ± 5.78		1.07 (1.01- 1.13)	**0.025**	1.12 (0.93–1.35)	0.244
Substance abuse noted at admission						
Yes	98 (35.25)	15 (5.40)	1.65 (0.76—3.57)	0.203	-	
No	151 (54.32)	14 (5.04)	1			
Alcohol						
Yes	53 (19.06)	5 (1.80)	0.77 (0.28 – 2.12)	0.613	-	
No	196 (70.50)	24 (8.63)	1			
Khat						
Yes	89 (32.01)	15 (5.40)	1.92 (0.89- 4.17)	0.097	3.59(1.21–10.65)	**0.021**
No	160 (57.55)	14 (5.04)	1		1	
Cigarette						
Yes	70 (25.18)	8 (2.88)	0 .97 (0.41—2.30)	0.952	**-**	
No	179 (64.39)	21 (7.55)	1			
Adherence to psychotropic medications						
Non-adherent	124(44.60)	20(7.19)	2.24 (0.98–5.11)	0.055	1.92 (0.67–5.59)	0.233
Adherent	125 (44.96)	9 (3.24)	1		1	
Is there ADR?						
Yes	45 (16.19)	10 (3.60)	2.39 (1.04- 5.48)	**0.040**	**2.39 (0.90–6.25)**	0.080
No	204 (73.38)	19 (6.83)	1		1	

## Discussion

This cross-sectional study assessed the management practice and discharge outcome of psychiatric patients admitted to psychiatry wards of selected hospitals in Ethiopia. Accordingly, in terms of pharmacotherapy, psychiatric polypharmacy was observed in a greater proportion, 232 (83.45%), of the study participants. This may be partly attributed to the patient’s presentation with an acute exacerbation of symptoms and, in some cases, a long-standing disease with different psychotropic medication trials. There are several reports indicating a high prevalence of psychiatric polypharmacy in recent clinical practice. Despite widespread use, there is relatively little evidence that this strategy is helpful, particularly when clozapine is not involved [31]. In patients with schizophrenia, augmentation treatment can be considered as a strategy to address initial nonresponse or partial response to antipsychotic treatment. Particularly for patients with negative symptoms or depression, augmentation of antipsychotic therapy with an antidepressant medication may be helpful. Use of a benzodiazepine, such as lorazepam, is also suggested in patients who exhibit catatonia. Combination therapy with two antipsychotic medications may reduce emergency room visits and readmission rates in patients receiving polypharmacy as compared with monotherapy. Moreover, there is no evidence that combining psychotropic medications is any more harmful than using a single medication, beyond the common side effects of each drug. However, it is important to consider whether factors are present that are influencing the treatment response towards antipsychotic monotherapy before considering augmentation treatment. Such factors may include concomitant substance use, rapid medication metabolism, poor medication absorption, interactions with other medications, and other effects on drug metabolism (e.g., smoking) that could affect blood levels of medication [32]. This implies that psychiatric polypharmacy should only be considered after ruling out the possible reasons for partial or non-response towards antipsychotic monotherapy after appropriate duration of treatment.

Regarding outcomes on discharge, the present study demonstrated high rates of improvement in symptoms of patients upon discharge (89.57%). The relatively longer hospital stay (40.9 ± 20.7 days) and clinician-observed medical therapy might have contributed to the higher rate of improvement in symptoms upon discharge. However, participants discharged without improvement should not be overlooked because this group of patients is at higher risk of early readmission and subsequent progression of a chronic condition. This finding is similar to studies from Ethiopia (90.3%) [12] and Nepal (92.2%) [15]. However, it’s higher than the reports of another study done in Ethiopia (74.9%) [17]. The deviation might be due to the study design (retrospective design in the previous study) and larger sample size (402 vs. 278) in other studies.

In this study, Khat users were about 3.6 times more likely to be discharged without improvement than patients who were non khat users. The possible explanation for this could be the increase in psychiatric symptoms among khat users during their admission and hospital stay. In one large meta-analysis [33], khat use was associated with a 122% increase in the prevalence of psychiatric symptoms. Another explanation could be khat use habit associated cognitive impairment, particularly poor decision-making might have contributed patients to discharge themselves against medical advice or absconded. A systematic review by Ayan A. et al. revealed that khat use was associated with cognitive impairments in different domains, including attention, cognitive flexibility, conflict resolution, decision-making, information processing speed, inhibitory control, learning, motor speed/coordination, short-term memory/working memory, and visual memory [34]. This implies that interventions targeted to reduce khat use among psychiatric patients might help to improve their discharge outcomes and to reduce the subsequent early readmission.

### Limitation of the study

The sample size used was relatively small compared to retrospective studies conducted using registries due to the short study period and resource constraints. Moreover, our study was not able to detect the effect of the specific past psychotropic medications the patients were taking, and we were dependent only on the general presence or absence of past psychotropic medication use history. Furthermore, the trigger tool used to detect adverse drug reactions was taken from an article and was not validated in Ethiopia.

## Conclusion

Psychiatry polypharmacy is a common treatment approach in patients with psychiatric disorders. The second-generation antipsychotics, especially risperidone, were commonly used for the treatment of psychiatric disorders along with other medications as combination therapy. Higher rates of improvement in symptoms were observed upon discharge. However, participants discharged without improvement should not be overlooked. Hence, interventions targeting risk factors, especially khat use, should be undertaken to improve their discharge outcome.

## Supplementary Information

**Additional file 1:** **Supplementary Table 1.** Dataset of the study ’Management practice and discharge outcome of patients with psychiatric disorder admitted to psychiatry wards of selected specialized settings in Ethiopia'. 

## Data Availability

All data generated or analysed during this study are included in this article (and its Supplementary information file).

## Abbreviations

ADR: Adverse drug Reactions
CGI: Clinical Global Impression
ECT: Electroconvulsive therapy
PWJMC: Psychiatry Ward of Jimma Medical Center
JMC: Jimma Medical Center
LOS: Length of Hospital Stay
MARS-5: Medication Adherence Rating Scale -5
SAMSH: St. Amanuel Mental Specialized Hospital
